# Pattern and Trend of Night Land Surface Temperature in Africa

**DOI:** 10.1038/s41598-019-54703-z

**Published:** 2019-12-04

**Authors:** Cherdchai Me-ead, Rhysa McNeil

**Affiliations:** 1 Faculty of Science and Technology, Prince of Songkla University, Pattani, Thailand; 2 Centre of Excellence in Mathematics, Commission on Higher Education (CHE), Ministry of Education, Ratchathewi, Bangkok, 10400 Thailand

**Keywords:** Statistics, Climate and Earth system modelling

## Abstract

This study aims to identify patterns and trends of the night land surface temperature over eight day period from 2000 to 2014 in Africa using statistical analysis. Data were obtained from the United States National Aeronautics and Space Administration satellite, comprising 99 locations of 5° by 5° latitude and longitude grid-boxes between latitudes 35° north and south of the equator and longitudes 20° west to 50° east. First, the variation in the night surface temperatures was removed. Then, the trend of seasonally adjusted night temperatures was estimated using linear regression. The correlations between adjoining regions were considered by using factor analysis to classify the temperatures into four regions. Cubic spline models were fitted to the data within these regions to investigate patterns of the temperatures. The result showed that temperatures in most regions of Africa increased. The temperatures decreased was observed in southern Africa and parts of central and eastern Africa.

## Introduction

Climate change is a major environmental problem affecting all countries. In recent years, increased temperatures have caused changes in sea levels, destruction of ecosystems, shrinkage of mountain glaciers, reduction of ice cover^1^ and altered ocean circulation patterns^2^. Moreover, climatic variability is associated with the El Niño Southern Oscillation and natural disasters^3^.

Numerous studies have shown variations in climate and associated changes in temperature in Africa^4–7^. Africa lies in tropical and subtropical latitudes; the temperatures are considerably high throughout the year and vary from day to night. In more than a third of the continent, the annual temperature range is less than 6 °C. Temperatures are relatively constant from year to year except in the highest latitudes. A cold season arose at the poleward extremes of the north of the Sahara and areas of southern Africa^6^. The continent of Africa has been warmer than 100 years ago. Global warming was about 0.5 °C over the 20 century^8^.

Several studies have investigated the trends of temperature in Africa. During 1960 to 1990, on average the maximum temperatures increased by 0.11 °C per decade and 0.12 °C per decade for non-urban and urban stations, respectively^9^. The average surface minimum and maximum air temperatures of 71 stations from 1939 to 1992, over eastern Africa were varied, mostly for the stations close to a large water feature^10^.

The climates of Africa range from humid equatorial regions to seasonally arid tropical regions and subtropical. The climates have differing degrees of temporal variability, with regard to rainfall. Assessing and estimating the spatial and temporal variation in temperature is crucial for climate science and for designing environmental programs^8^. This study considers night time observations to reduce the scale mismatch issue between point ground measurements and the MODIS footprint. A series of satellite pixels of Africa can be used to provide the data of temperature changes of the region, both days and nights. During the night, the earth’s surface is quite homogeneous surface. During the day, surface temperatures under shadows are lower than surface temperatures in direct sunlight, the temperature differences of about 20 °C^11^. Therefore, with this view and importance, this study investigated the trends and patterns of night land surface temperatures over eight day periods from 2000–2014 in Africa using appropriate statistical methods.

## Materials and Methods

### Data management and study areas

The study area is between latitudes 35° north and south and longitudes 20° west to 50° east, covering the entire African continent. This study used Moderate Resolution Imaging Spectroradiometer (MODIS) data which observed by NASA’s satellite. MODIS measures climate variables such as land surface temperature on and above the Earth’s surface by decoding solar reflectance images viewed by an Earth-orbiting satellite.

**Figure 1:**
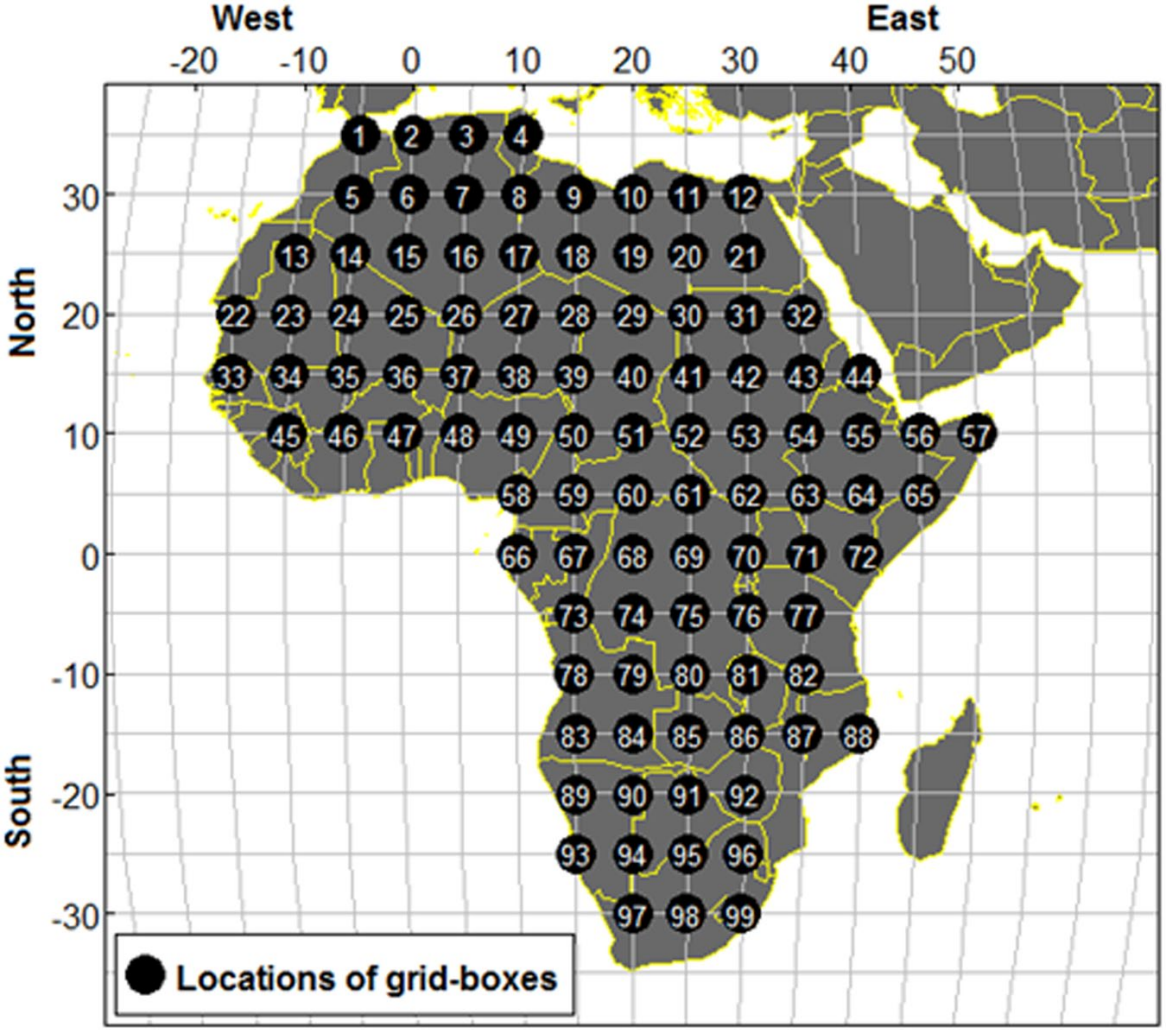
Map of the study area showing 99 locations of satellite image by NASA satellite (5 latitude by 5 longitude).

The night time land surface temperatures were stored as integers. MODIS is used to capture pictures of the Earth overtime at the same location. It records satellite pixels for every eight days and provided them publicly between 2000 to 2014. These pixels can be converted into temperatures from their color index. The temperatures are divided by 50 to obtain degrees Kelvin^12^, and subtracting 273.15 to obtain the equivalent degrees Celsius. Zeroes relate to missing data due to insufficient measurement quality. This can occur when water vapor and/or cloud are in the line of sight. The data has 668 records corresponding to the days when the satellite recorded images for this region. The period between each such observation is slightly less than eight days because each year has 46 observations for the same region. The series of satellite pixels of Africa can be used to provide the data of temperature changes of the region. The night temperature data for 15 years were obtained from the website (http://daac.ornl.gov/cgi-bin/MODIS/GLBVIZ_1_Glb/modis_subset_order_global_ col5.pl) for 99 point locations of 5° by 5° latitude-longitude grid-boxes (81 square kilometers per point location) in Africa. Thus, there were 99 point locations for the analysis as shown in Figure 1.

### Statistical methods

The variation in temperatures for every eight day period was removed by subtracting the average temperature in each period and adding back the overall mean of the 99 locations. It takes the form:

1 $${y}_{ij}=({x}_{ij}-{\bar{x}}_{\cdot j})+{\bar{x}}_{\cdot \cdot },$$

where *y*_*ij*_ is the seasonally adjusted night land surface temperatures for every eight day period at year *i* for period *j*, *x*_*ij*_ is the night land surface temperatures for every eight day period at year *i* for period *j*, ${\bar{x}}_{\cdot j}$ is the average of period *j* over 15 years and ${\bar{x}}_{\cdot \cdot }$ is the overall mean.

A linear regression method^13^ is arguable one of the most widely used parametric test in trend detention in climate science. The application of linear regression in trend analysis includes sea surface temperature trends^14^ and rainfall trends^15^ was applied to the seasonally adjusted night surface temperature changes in an eight day period for each of the 99 locations in Africa. The model takes the form:

2 $${y}_{ijk}={m}_{k}+{a}_{ik}+{b}_{jk},$$

for *i* = 1, 2, 3,…, 15, *j* = 1, 2, 3,…, 46 and *k* = 1, 2, 3,…, 99 where *y*_*ijk*_ denotes the seasonally adjusted night surface temperature of the eight day period in the year *i* for period *j* and at location area *k*, *m*_*k*_ is a constant for location area *k*, and the terms *a*_*ik*_ and *b*_*jk*_ are estimated coefficients for year *i* and period *j*, respectively.

The night land surface temperatures in the 99 locations were grouped into regions to account for the correlation between adjoining regions using factor analysis^16^. Factor analysis is arguably one of the mostly used method to reduce a set of correlated variables into a smaller set of uncorrelated variables by combining variance in highly mutually correlated subgroups, preferably with minimal loss of information^14,15^. The model with *p* factors takes the form:

3 $${y}_{kj}={\mu }_{k}+\mathop{\sum }\limits_{p=1}^{m}{\lambda }_{kp}{\varphi }_{kp},$$

where *k* = 1, 2, 3,…, 99, *j* = 1, 2, 3,…,46 and *p* = 1, 2, 3,…, *m* and *y*_*kj*_ is the average land surface temperature in an eight day period at location *k* for period *j*; *µ*_*k*_ is the average across 46 months for the location *k*; *λ*_*kp*_ is the factor loadings at *k*^*th*^ location on *p*^*th*^ factor; and, *ϕ*_*kp*_ is *p*^*th*^ common factor for location* k*.

This information was expressed as the proportion of variance in the data accounted for by the factor analysis. A set of loadings gives measures of the extent to which each of the original variables correlates with each factor. The loadings (usually between −1 and 1) were controlled using Promax rotation, to make these loadings as close to 0 or 1 as possible. The loading factors of more than 0.33 were used as the criteria to classify regions in each factor^17^. A spline function is a piecewise cubic polynomial with continuous second derivatives, and is smoothest among all functions in the sense that it has minimal integrated squared second derivative. It is fitted using linear least squares regression. The end of every year is followed by the beginning of the next year and so the model is a smooth periodic function within the annual connection points for the whole 15 year period. The connection points are known as knots. The most appropriate model is a cubic spline with boundary conditions ensuring smooth periodicity. The formula for cubic spline function takes the form^18,19^:

4 $$S(t)=a+bt+\mathop{\sum }\limits_{r=1}^{q}{c}_{r}{(t-{t}_{r})}_{+}^{3},$$

where, *S*(*t*) is the spline function of the data, *a, b* and *c* are the constants. *r* is the locations of the knots starting from 1, *q* is the total number of knots, *t* denotes time, *t*_1_ < *t*_2_ < … < *t*_*q*_ are specified knots and (*t* − *x*)_+_ is *t* − *x* for *t* > *x* and 0 otherwise. An advantage of the use of cubic spline function in modelling is its ability to handle a significant amount of missing data.

Graphical and statistical displays were created using R^20^.

## Result and Discussion

**Figure 2:**
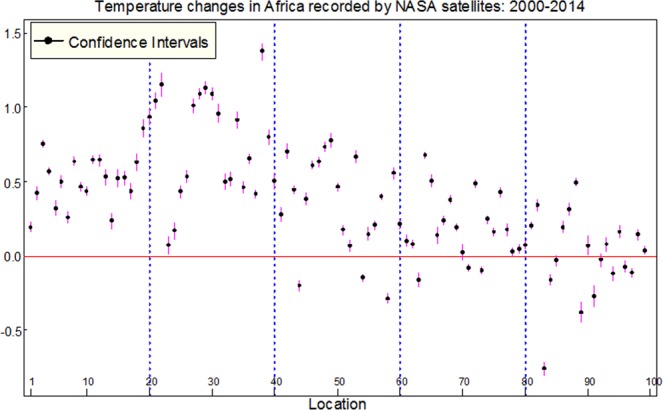
Estimates and 95% confidence intervals for land surface temperature changes at night in Africa from 2000–2014.

The results of linear regression model provided temperature changes and their 95% confidence interval for 99 locations in Africa as displayed in Figure 2. Eighty-one locations showed a significant temperature increase, 12 locations show a significant decrease and 6 locations showed no change. The maximum increase in temperature was observed at latitude 15°N and longitude 10°E with an increment of 1.38 °C and the minimum increase was observed at latitude 10°S and longitude 15°E and also at latitude zero and longitude 30°E with an increment of 0.03 °C. The maximum decrease in temperature was found at latitude 15°S and longitude 15°E with the decrement of 0.76 °C, while the minimum decrease in temperature was found at latitude 20°S and longitude 30°E and also at latitude 15°S and longitude 25°E with a decrement of 0.03 °C, respectively. Assessment of the autocorrelation graphs did not show any significant serial correlation, which might revealed worse of fit of the models.

**Figure 3:**
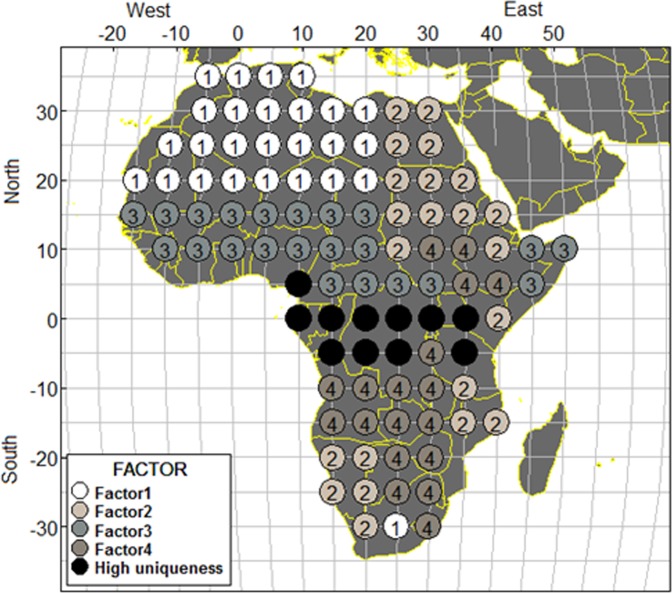
Factor analysis classifying the 99 locations into 4 regions. Numbers in the circles represent the factor number.

The correlation between night temperatures at eight day intervals between pixels 1 km apart in the 99 locations ranged from 0.00004 to 0.89948. Factor analysis classified the temperatures into regions (factors) by considering the value of the factor loadings. Table 1 shows the factor loadings in ascending order within each factor and uniqueness value. Four factors were identified. Factor 1 consists of 26 locations, factor 2 consist of 22 locations, factor 3 consist of 17 locations and factor 4 consist of 16 locations. Seven locations (locations 38, 39, 43, 53, 56, 57 and 95) had mixed factors, while 18 locations were considered unique to others. Thus, region with uniqueness value above 0.78 and loading below 0.3 and negative loading were omitted from further analysis. Figure 3 displays a map of Africa and the four regions as identified by the factor model. The 99 locations are represented by the circles and coded from factor 1 to factor 4. Eleven locations are represented by an empty circle (darkest shade) representing high uniqueness regions and could not be classify into any region. This model accounted for 48.4% of the total variance in the night temperatures.

**Table 1:** Factor loading scores from factor analysis.

Temp	Factor1	Factor2	Factor3	Factor4	Uniqueness
7	**0.97**	−0.12			0.16
14	**0.97**				0.18
4	**0.96**				0.22
6	**0.96**	−0.12			0.16
15	**0.96**				0.17
5	**0.95**	−0.11			0.17
8	**0.95**				0.17
3	**0.95**	−0.10			0.19
9	**0.95**				0.19
13	**0.93**				0.24
2	**0.91**				0.27
16	**0.91**				0.17
17	**0.89**				0.17
10	**0.88**				0.25
1	**0.85**				0.33
23	**0.84**				0.27
18	**0.83**				0.19
24	**0.83**		0.11		0.24
19	**0.74**		0.16		0.22
25	**0.64**		0.25		0.32
29	**0.63**		0.25	−0.11	0.25
28	**0.63**		0.26	−0.13	0.23
27	**0.63**		0.27	−0.11	0.24
26	**0.61**		0.28		0.28
22	**0.58**		0.21		0.46
98	**0.46**		−0.20	0.33	0.33
21	−0.15	**0.98**			0.14
20	−0.15	**0.98**			0.14
30	−0.15	**0.96**			0.19
31	−0.15	**0.93**			0.23
11	−0.12	**0.92**			0.19
12	−0.13	**0.91**			0.20
41		**0.80**		−0.12	0.40
42	−0.13	**0.79**			0.41
94		**0.75**			0.40
32		**0.74**			0.44
97		**0.74**		−0.14	0.40
90	−0.16	**0.63**		−0.10	0.53
89	−0.10	**0.62**		−0.22	0.53
88	−0.11	**0.59**		−0.17	0.59
44		**0.58**	−0.13	−0.11	0.64
52	0.12	**0.55**	−0.12	−0.24	0.59
82	−0.10	**0.53**			0.68
93		**0.51**		−0.28	0.60
72	−0.15	**0.45**		−0.42	0.57
87	−0.28	**0.42**		−0.14	0.69
43		**0.38**	−0.19	−0.34	0.76
55	0.13	**0.35**		−0.29	0.75
50			**0.73**		0.47
49			**0.71**	−0.12	0.45
36		0.13	**0.70**		0.42
48	−0.11		**0.69**		0.60
34			**0.67**		0.53
47			**0.66**		0.61
35	0.13		**0.65**		0.46
51			**0.64**		0.54
40	0.11		**0.64**	−0.13	0.44
46			**0.63**		0.64
33			**0.61**		0.61
37	0.23	0.13	**0.60**		0.39
45			**0.56**		0.67
38	0.35	0.11	**0.48**	−0.13	0.35
39	0.39		**0.45**	−0.13	0.35
57	0.37		**0.40**	−0.13	0.44
56	0.38		**0.39**		0.47
85				**0.78**	0.40
86		0.13		**0.74**	0.44
84				**0.74**	0.43
80			0.13	**0.68**	0.62
79				**0.68**	0.59
83			−0.11	**0.67**	0.51
92	0.11		−0.18	**0.64**	0.38
91	−0.13		−0.17	**0.63**	0.38
81	−0.10		0.12	**0.63**	0.56
96	−0.22		−0.22	**0.52**	0.40
78	−0.16		0.19	**0.50**	0.68
54				**0.49**	0.70
76	−0.10		0.17	**0.46**	0.75
53		0.35		**0.43**	0.71
95	0.37		−0.20	**0.42**	0.34
99	−0.29		−0.13	**0.42**	0.51
70		0.11	0.12		0.97
68		−0.12	0.13		0.96
69	−0.15		0.21		0.96
66	−0.22	−0.12	0.11		0.94
58	−0.20				0.93
67	−0.25		0.22		0.93
74				−0.30	0.92
75			0.16	0.29	0.91
60			−0.41		0.87
63	−0.13			−0.41	0.87
62	−0.26	−0.12	−0.36	0.13	0.83
59	−0.12		−0.46		0.82
71		−0.30		−0.25	0.82
73	−0.21		0.19	−0.31	0.82
65		−0.14	−0.35	0.26	0.82
61	−0.13		−0.50		0.81
64	−0.21	−0.12	0.10	−0.48	0.79
77	−0.30	−0.29	−0.12	−0.11	0.78

After the 99 locations were categorized into larger regions using factor analysis, the time series plot was used to display the temperature pattern in the 4 factor regions. We found that most factors were not situated in the same region. For example, 25 locations representing factor 1 was found between latitudes 20°–35° north while only one location in factor 1 was found in latitudes 30° south. The same applies to factor 2 as well as 13 locations between latitudes 10°–30° north, one location in the equator and 8 locations between latitudes 10°–30° south.

**Figure 4:**
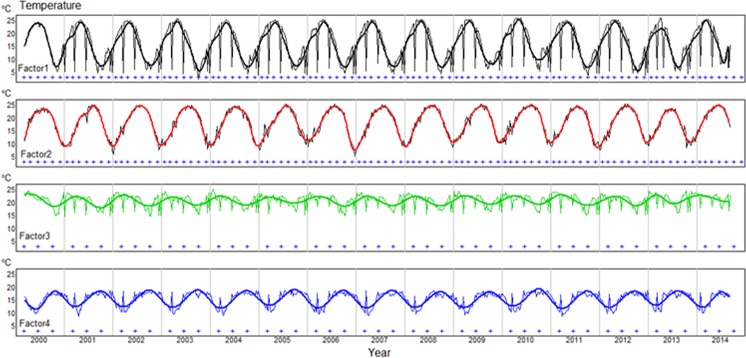
Time series plots of night land surface temperatures for four regions in Africa. The + symbols represent cubic spline knots.

All factors were displayed using time series plots as shown in Figure 4. Natural cubic spline functions were used to fit the temperature data in each factor. To fit the cubic spline to these data the knots were selected for the spline functions at seven times during each year for factors 1 and 2, and at three times per year for factors 3 and 4 (denoted by + symbols in the graphs). The plots of all factors display clear periodic variation patterns, with peaks at different months of the year. The pattern of temperature in factor 1 was similar to factor 2 and showed higher volatility, especially for factor 1 which had peaked in May and troughs in October in every year. Furthermore, factor 3 was similar to factor 4 and showed a lower variation of temperature, which might reflect climatic variation. In factor 1 and 2, 7 knots equally spaced in each year were found to be appropriate fit. However, in factor 3 and 4, 3 knots equally spaced in each year were found to be appropriate fit. The number of knots to fit the data could be dependent on the variation of the temperature. More variation of data will require the use of more knots for proper fit and less variation will require the use of fewer knots.

The findings of this study show evidence of temperature variation in the African continent from 2000 to 2014. Temperature changes in the north of Africa were higher than those in the south, These higher changes were mainly in the higher latitudes. This finding was consistent with previous studies^21^. Another study indicated that the northern part of Africa had night time warming in recent years^10^. Moreover, the land areas over the Sahara and semi-arid parts of southern Africa warmed by 1.6 °C up to 2050 s and the equatorial African countries warmed at a steady rate of about 1.4 °C per year^22,23^. The maximum temperatures over Zimbabwe for the period 1897 to 1993 showed a warming trend during all seasons^24^. The rising temperatures observed in the land areas over the Sahara, semi-arid parts of southern Africa and the equatorial African countries may be detrimental to the life of some crops. An increasing temperature may cause yield declines between 2.5% and 10% across a number of agronomic species throughout the 21st century^25^. Other investigations on the evaluations of temperature on crop yield have reported varying results, estimates of yield decline between 3.8% and 5%^26^.

Temperatures decreased was observed in southern Africa, parts of central Africa, and parts of eastern Africa. The trends of the drop in temperature were also consistent with a other studies^10,24^, the minimum temperatures over Zimbabwe for the period 1897 to 1993 had no trend or a slight decrease. South African maximum temperatures had decreased from 1940 to 1989^27^. In our study eighteen locations in central Africa and eastern Africa had both increases and decreases in temperature. The temperatures in these locations are typical of equatorial regions and are influenced by a combination of the region’s high altitude and the rain shadow of the westerly monsoon winds.

## Conclusions

Night land surface temperatures for every eight day periods data were studied from 2000 to 2014 for each of 99 locations of 5° by 5° latitude-longitude grid-boxes in Africa. Statistical methods comprising linear regression models, time series analysis, factor analysis, and cubic spline were used to investigate the features and classify temperature variability in this study. Analysis of night land surface temperatures for every eight day period found that the temperature increase in 81 locations, 12 locations showed decrease temperature and 6 locations showed no change. The temperature changes ranged from −0.76  °C to 1.38 °C. The maximum increase in temperature, approximately 1.38 °C, occurred in the desert area of the North of Africa. The minimum increase temperature, approximately 0.03 °C, occurred in the East of Africa. Factor analysis showed diverse patterns of temperature in the four factors. Natural cubic spline functions were fitted to display the variation pattern in each factor. Even though the observed 15 years temperature trends may not imply climate change, the observe trends and patterns is essential to various stakeholders particularly the agronomist in the planning of the various crop operations.

In this study, only night time observations were investigated in Africa which are considered a critical areas for global warming. Further studies could explore the daytime temperature. We have employed only temperature data without taking into account other factors affecting the variation of temperature. Consequently, different areas and the relationship between the temperature and the other climate factors should also be investigated. The study related land surface temperature changes to land use could also investigate. The techniques presented in this research can be extended to other studies such as rainfall, wind, and solar radiation.
